# Genome-wide identification and integrated analysis of the *WNK* kinase gene family and expression analysis of *PgWNK* genes under methyl jasmonate treatment in *Panax ginseng*

**DOI:** 10.1186/s12870-025-06818-x

**Published:** 2025-07-01

**Authors:** Kexin Zhang, Aimin Wang, Lin Shi, Jiaqing Liu, Yu Zhang, Yang Jiang, Meiping Zhang, Yi Wang, Mingzhu Zhao, Kangyu Wang

**Affiliations:** 1 College of Life Science, Jilin Agricultural University, Changchun, Jilin, 130118 China; 2 Jilin Engineering Research Center Ginseng Genetic Resources Development and Utilization, Jilin Agricultural University, Changchun, Jilin, 130118 China

**Keywords:** *Panax ginseng*, *WNK* (with no lysine) kinase gene family, Protopanaxatriol-type ginsenosides, Methyl jasmonate (MeJA) treatment, Ginseng adventitious roots

## Abstract

Ginseng (*Panax ginseng*) is a globally renowned medicinal plant. The primary active compounds in ginseng are ginsenosides, which have been shown to possess preventive and therapeutic properties against a range of ailments. WNK (with no lysine) kinases is a subfamily of serine/threonine protein kinases. Members of the *WNK* gene family play vital roles in the regulation of plant growth, development, and biological processes. Several studies have shown that jasmine methyl ester treatment can increase the content of protopanaxatriol-type ginsenosides in adventitious ginseng roots. However, there are no reports on the effects of *WNK* in ginseng under methyl jasmonate (MeJA) treatment. In this study, we conducted a detailed screening, identification, and systematic research on the *WNK* gene family in ginseng, including phylogenetic relationships, gene structure, chromosomal distribution, GO functional classification, cis-regulatory elements, co-expression network analysis, and gene expression pattern analysis. Furthermore, we identified candidate genes of *PgWNK* that could significantly respond to methyl jasmonate regulation and conducted an in-depth study of their expression patterns to explore the impact of these candidate genes on the variation in triterpenoid ginsenoside content. We identified 32 *WNK* genes in ginseng that were unevenly distributed across 11 of the 24 chromosomes in the ginseng genome. GO functional annotation and enrichment analysis of the *PgWNK* genes revealed their involvement in numerous biological processes, including ginseng growth, development, and biosynthesis of secondary metabolites. Through expression network analysis, we further understand that there are complex interactions among members of the *PgWNK* gene family. In addition, 17 non-biological stress response components related to hormone response, growth, and development were predicted in the promoter area of the *PgWNK* gene. At the same time, we studied the gene expression of *PgWNK* after MeJA treatment and found that the expression related to ginsenoside biosynthesis was significantly increased as the regulation time was prolonged. Following MeJA regulation, the content of five protopanaxatriol-type ginsenosides (Re, Rf, Rg1, Rg2, and Rh1) increased owing to the adjustment of *PgWNK05* gene expression. Therefore, our results provide an important theoretical foundation for understanding the molecular characteristics, genetic evolution, genetic functions, expression patterns, and non-biological coercion of the *WNK* gene family in ginseng. The underlying research on the biological synthesis of ginsenosides provides a foundation and theoretical support.

## Introduction

WNK kinase is a unique serine/threonine protein kinase [1] composed of an N-terminal kinase domain and a C-terminal regulatory domain. The kinase subdomain II in the N-terminal kinase domain lacks a catalytic lysine residue; hence, the name WNK (with no K, i.e., lysine) [2]. This lysine residue is conserved in all other kinases and is crucial for ATP coordination at the active center (Huang et al. 2007). In most kinases, this lysine residue is important for maintaining protein kinase activity because it interacts with the α and β phosphate groups of ATP, promoting ATP binding for substrate phosphorylation and kinase activation [3]. In plant WNK kinases, these are replaced by asparagine/serine/glycine residues. Despite these amino acid substitutions, WNK kinases remain functional and are involved in various plant physiological processes [2]. Although WNK kinases have been reported to affect the growth and development processes of many plants [4], for example, by regulating the circadian rhythm, vacuolar H^+^-ATPase and photoperiod pathways to regulate the flowering time of *Arabidopsis thaliana* [5], the soybean transcriptome database reveals the tissue-specific and salt stress-responsive expression of soybean *WNK* genes [6], the *OsWNK1* gene family in rice responds differently to various abiotic stresses such as cold, high temperature, salt, and drought [7], and the function of the *WNK* gene family in regulating the circadian rhythm in upland cotton has been well studies [8]. However, there are no reports on the functional role of the *WNK* gene family in ginseng.

Ginseng (*Panax ginseng*), a perennial herb of the Araliaceae family [9], is mainly distributed in China, Russia, and North Korea, and its medicinal value can be traced back to five thousand years ago [10]. The main secondary metabolite of ginseng is ginsenoside [11], a class of triterpenoid compounds linked to carbohydrate chains that are the main active components of ginseng [12]. The diversity in the structure and function of ginsenosides is related to aglycones [13]. Ginsenosides are categorized as tetracyclic triterpenoid dammarane-type saponins [14], pentacyclic triterpenoid oleanolic acid-type saponins, and oleanolic acid-type ginsenosides based on their aglycone structures [15]. Dammarane-type ginsenosides are further subdivided into protopanaxadiol-type (PPD) and protopanaxatriol-type (PPT) ginsenosides [16]. To date, over 200 ginsenoside components have a variety of pharmacological activities, including antioxidant, anti-inflammatory [17], vasodilation [18], anti-allergy [19], and anti-diabetic [20], and have pharmacological effects such as inhibiting cancer cell proliferation [21], treating myocardial ischemia and inhibiting central nervous system oxidation [22]. Although there are many types of ginsenosides, owing to their complex structure and low content of rare ginsenosides, it is difficult to extract and separate them, and they cannot be fully studied and utilized [23]. Therefore, it is particularly important to use biotechnological methods to improve the ginsenoside content in ginseng [24].

Induction agents are divided into biological and abiotic inducers that can cause changes in secondary metabolic pathways or metabolic intensity [25]. Their main function is to regulate the activity of certain enzymes in secondary metabolic pathways and regulate or activate specific genes in secondary metabolic pathways at the transcriptional level, causing changes in the intensity of secondary metabolism, thereby regulating the content of secondary metabolites [26]. Methyl jasmonate (MeJA), an abiotic inducer, plays an important role in regulating the biosynthesis of ginsenosides in ginseng, whether it is a change in endogenous JAs produced by plants themselves under adverse conditions or treatment with exogenous JAs [27]. The addition of exogenous MeJA can regulate the triterpenoid saponin content in ginseng adventitious roots and the expression of genes related to triterpenoid biosynthesis [28]. Therefore, this study employed methyl jasmonate (MeJA) as an exogenous elicitor to chronologically treat ginseng adventitious root cultures, thereby investigating the temporal dynamics of *PgWNK* gene expression and its regulatory roles in ginsenoside biosynthetic pathways across distinct chronological treatment intervals.

This study employed an integrated to systematically characterize *WNK* gene family in *P. ginseng*, utilizing genomic and transcriptomic datasets for comprehensive identification. And phylogenetic relationships, gene structure, chromosome distribution, GO functional classification, cis-acting elements, co-expression, and gene expression patterns were analyzed, and it was found that 20 *PgWNK* genes were found to contain rich methyl jasmonate (MeJA) response elements. After treatment of ginseng adventitious roots with 200 mM MeJA, the expression patterns of five *PgWNK* genes at different treatment times were examined, and it was found that the content of five protopanaxatriol-type saponins (Re, Rf, Rg1, Rg2, and Rh1) increased with the upregulation of *PgWNK* gene expression. The present study provides essential insights into *WNK* genes of ginseng and lays the foundation for more in-depth experimental exploration of the exact functions of these candidate *WNK* genes.

## Materials and Methods

### Ginseng samples and transcriptome databases

In this study, four different age stages (5, 12, 18, and 25 years), 14 different tissues of four-year-old ginseng (stem, fiber root, fruit peduncle, main root epidermis, fruit pedicel, rhizome, leaf peduncle, arm root, leaflet pedicel, leg root, leaf blade, fruit flesh, main root cortex, and seed), and 42 local cultivars of ginseng roots (S1 – S42) were collected from Jilin, China (late July 2010). The datasets analyzed during the current study are available in the four different age stages, 14 different tissues, and 42 local cultivars of Jilin* P. ginseng* transcriptome databases. All ginseng plant materials were stored in Jilin Engineering Research Center Ginseng Genetic Resources Development and Utilization, Jilin Agricultural University.

### Identification of the *WNK* gene family in ginseng

Leveraging genomic and transcriptomic databases of ginseng, we implemented HMMER software for systematic mining of WNK domain through whole-genome protein alignment (E-value of 1 × 10⁻⁶). Following initial identification, candidate sequences underwent rigorous domain architecture verification using NCBI's Conserved Domain Database and SMART online tools, ensuring that the candidate transcripts contained the conserved WNK domain and named them.

### Phylogenetic analysis, conserved domain and motif analysis of the *WNK* gene family

To classify the WNK kinase protein family in ginseng, the *PgWNK* kinase genes with complete ORFs were compared with those of the other five species. Sunflower (*Helianthus annuus*), a dicotyledonous plant; rice (*Oryza sativa*), a monocotyledonous plant; and *Acorus calamus*, along with the model plant *Arabidopsis thaliana* and the spore plant *Marchantia polymorpha* from the liverwort family, were selected as outgroup species. The 24 *WNK* genes of the five outgroup species were used for phylogenetic analysis of the *PgWNK* genes of ginseng. The maximum likelihood (ML) method in MEGA version X was used to construct the phylogenetic tree, with bootstrap repetitions set to 2000 [29]. The phylogenetic tree was constructed using the online website Evolview version 3.0 (https://www.evolgenius.info/evolview/#login), and conserved motif analysis was performed using MEME motif search tool (http://meme.nbcr.net/meme/), with the maximum and minimum conserved motif lengths being 10 and 50 amino acids, respectively.

### Chromosome localization and collinearity analysis of the ginseng *WNK* gene family

The *WNK* gene family was aligned with the ginseng genome using BLASTEN with alignment standards of identity ≥ 95%, coverage length ≥ 300 bp, and E-value ≤ 1.0E-100. The MG2 C online tool (http://mg2c.iask.in/mg2c_v2.1/index.html) was used to visualize the location of the *WNK* on genome chromosomes. Genomic synteny analysis of *WNK* genes homologs within the ginseng genome was followed by chromosomal mapping of *PgWNK* paralogs exhibiting tandem duplication patterns using R language of genomic visualization frameworks.

### *PgWNK* genes GO functional annotation and classification

We used Blast2GO version 6.0.3 to annotate and classify the identified *WNK* transcripts in GO [30], and used the EVeen online tool (http://www.ehbio.com/test/venn/#/) for visualization and analysis [31]. The results of annotation and GO classification were used to evaluate the functional differentiation of the *WNK* genes and the number of *WNK* transcripts involved in specific and multiple functions. The functions annotated at level 2 of the *WNK* transcripts were visualized using R language.

### Expression analysis of the *WNK* gene family in ginseng

*PgWNK* genes was conducted through systematic expression in the roots of ginseng at four different age stages, 14 different tissues of four-year-old ginseng, and 42 local cultivars of ginseng roots were extracted from the ginseng transcriptome expression database. Transcripts with zero expression were removed, and the data were visualized using the R language.

### Co-expression network analysis of *PgWNK* genes

To explore the interaction characteristics of *PgWNK* gene expression among the 42 local ginseng cultivars, Spearman’s correlation coefficient was calculated using SPSS version 23.0, and the co-expression network of *PgWNK* genes was constructed using BioLayout Express 3D version 3.3 software.

### Promoter cis-acting element analysis of the WNK gene family in ginseng

To elucidate potential biological functions of *PgWNK* genes, we conducted cis-regulatory element analysis on 2,000 bp promoter regions upstream of translation initiation sites, anchored by chromosomal coordinates. Sequences were computationally interrogated through the PlantCARE (https://bioinformatics.psb.ugent.be/webtools/plantcare/html/) for phytohormone-responsive cis-elements, followed by spatial mapping of identified motifs using TBtools II software [32].

### Identification of *WNK* candidate genes related to ginsenoside synthesis in ginseng

The expression data of *PgWNK* genes in 42 local cultivars of Jilin ginseng and the expression data of 20 key enzymes related to ginsenoside synthesis were organized, and the correlation between *PgWNK* gene expression and key enzyme-encoding gene expression was calculated using SPSS version 23.0 software for Pearson’s correlation coefficient analysis. The data were visualized using the Prism online software.

### Expression analysis of *PgWNK* genes in ginseng adventitious roots under MeJA treatment

Ginseng adventitious roots (1.0 g FW) were aseptically transferred to 250 mL Erlenmeyer flasks containing MS liquid medium (150 mL) and cultured under continuous orbital agitation (110 rpm) at 22 °C for 21 days in darkness. Ginseng adventitious roots were treated with 200 mM MeJA (Sigma Company, USA), an optimal concentration in the laboratory. Therefore, on the 22nd day, 200 mM MeJA was added to the culture bottle and the treatment times were 0, 6, 12, 24, 36, 48, 60, 72, 84, and 96 h. Three biological replicates were collected, and the 0 h group was used as the blank control. After a cultivation period of 30 days, the samples were collected. Then, 0.1 g of sample material were collected at different time points after MeJA treatment, and total RNA was extracted from the ginseng adventitious root tissue using the Trizol method. Total RNA was reverse-transcribed into cDNA using the RevertAid™ first-strand cDNA Synthesis Kit (MBI Company, USA), and *Actin 1* gene as the internal reference gene. The Applied Biosystems 7500 Real-Time PCR System with optimized cycling parameters. Reactions were configured as follows: 10 μL total volume containing 5 μL UltraSYBR mix (CWBIO, Beijing, China), 0.2 μL each of forward/reverse primers (10 μM), 1 μL cDNA template (50 ng/μL), and 3.6 μL ddH_2_O, following manufacturer's protocols for one-step reverse transcription amplification. The PCR cycle parameters were as follows: pre-denaturation at 95 °C for 10 min; 40 cycles of denaturation at 95 °C for 15 s, annealing at 60 °C for 60 s, and extension at 72 °C for 30 s. Three biological replicates and three technical replicates were used to ensure the accuracy of each treatment. The results were obtained using the 2^−ΔΔCt^ method.

### Correlation analysis between *PgWNK* gene expression and protopanaxatriol-type ginsenoside content under MeJA treatment

Independent sample *t-*test and one-way analysis of variance were used to analyze the differences in the five protopanaxatriol-types ginsenosides content Re, Rf, Rg1, Rg2, and Rh1 in 42 ginseng farmer’s cultivars as phenotypic data. A horizontal comparison and analysis were conducted on the expression of *PgWNK* genes and the content of ginsenosides induced by methyl jasmonate, and the correlation between *PgWNK* gene expression and the four ginsenosides Re, Rf, Rg1, Rg2, and Rh1 produced after MeJA treatment was statistically analyzed.

## Results

### Identification of the *WNK* gene family transcripts in *P. ginseng*

A comprehensive analysis of the genomic and transcriptomic database revealed 32 transcripts encoding WNK domains in* P. ginseng*. These sequences were systematically classified into either the PKc or sTKC superfamilies and designated as *PgWNK* gene followed by a numerical identifier (e.g., *PgWNK01*—*PgWNK32*). Detailed characterization of these transcripts, including gene/transcript IDs, sequence lengths, mRNA sequences (5'- 3'), open reading frame (ORF) dimensions (bp/aa), and conserved domain architectures, is comprehensively presented in Supplementary Table S1. Among these sequences, the shortest gene fragment *PgWNK09-14* was 200 base pairs (bp) in length, while the longest five gene fragments (*PgWNK09-03*, *PgWNK09-04*, *PgWNK09-09*, *PgWNK09-11*, and *PgWNK09-12*) were all 3312 base pairs (bp) in length, and the length of the encoded amino acids ranged from 200 amino acids (aa) to 1103 amino acids (aa).

### Phylogenetic analysis of the *WNK* gene family in ginseng

**Fig. 1:**
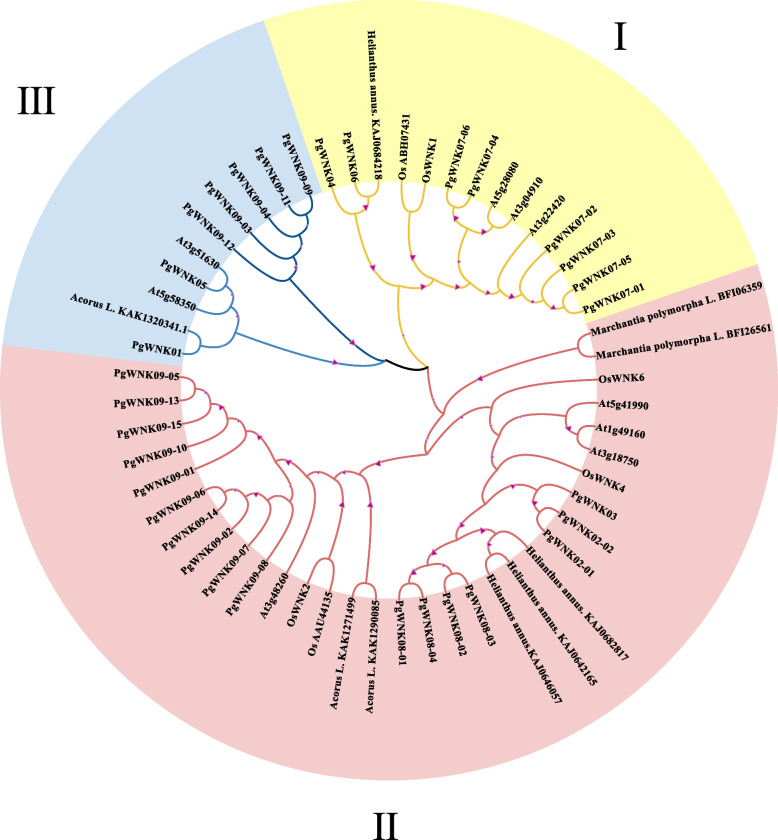
The phylogenetic tree of the *PgWNK* gene family with exogenous species. Transcripts of *PgWNK* with complete ORFs in ginseng are used as the outgroup, and 24 genes from *Arabidopsis thaliana* (*At*), *Oryza sativa *(*Os*), *Acorus calamus*, *Marchantia polymorpha*, and *Helianthus annus* are used as the outgroup. Subfamilies of the* PgWNK* gene family are denoted by I, II, and III

To reveal the evolutionary relationship of the *WNK* gene family in ginseng and classify it, the 32 *PgWNK* kinase genes with complete ORFs were compared with 24 *WNK* genes of five outgroup species, and the phylogenetic analysis was performed between the 24 *WNK* genes of the outgroup species and the *PgWNK* genes, and then the phylogenetic tree of the ginseng *WNK* kinase protein was constructed. As depicted in the Fig. 1, members of the *WNK* gene family can be categorized into three distinct branches, labeled I, II, and III, based on their phylogenetic relationships. Among these, branch III had the fewest members, comprising only seven members of the ginseng *PgWNK* gene family, whereas branch I had the largest number, with 17 members of the *PgWNK* gene family.

### Motif prediction and conserved structural domains of the *WNK* genes

**Fig. 2:**
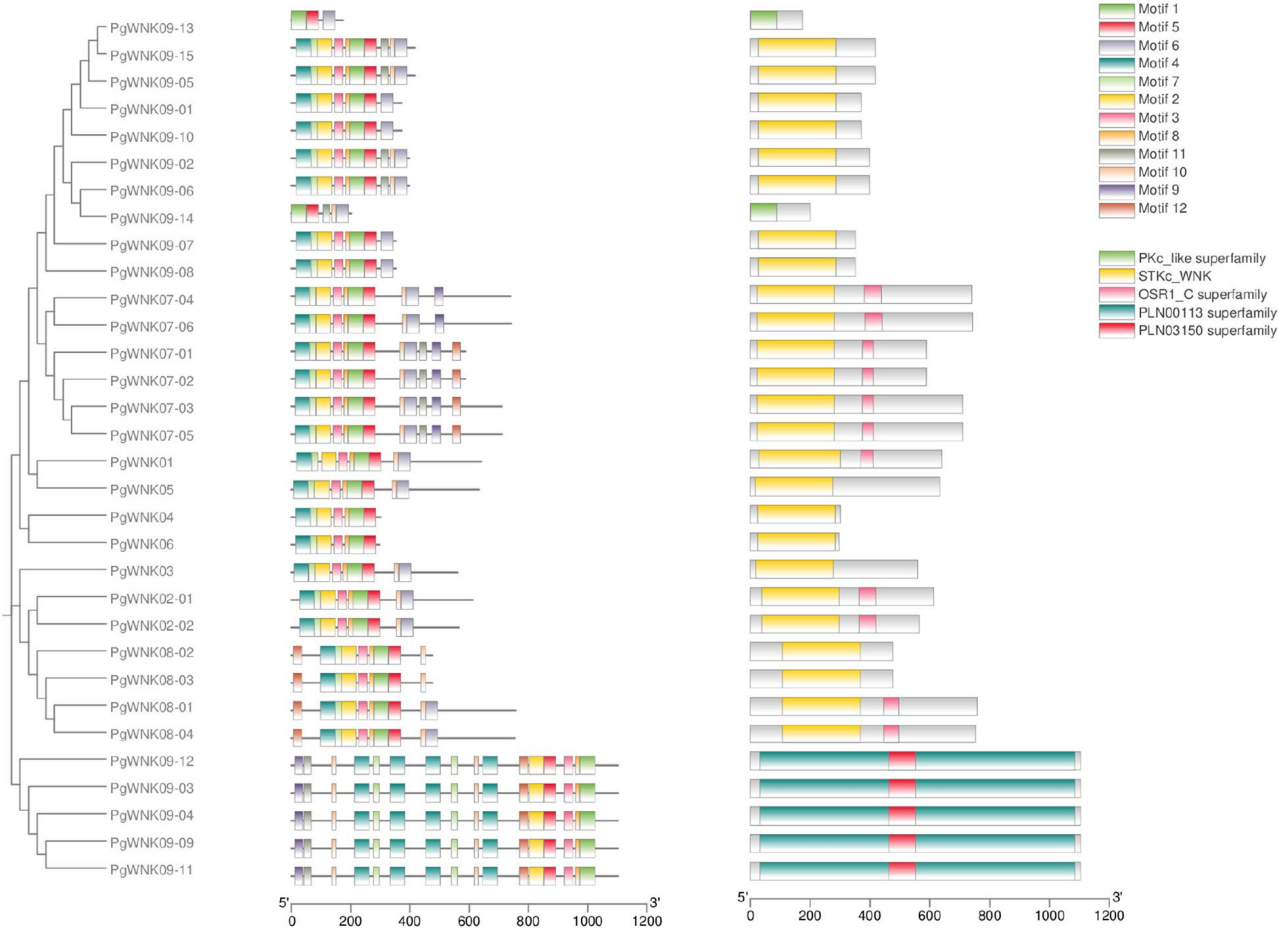
Phylogenetic tree, conserved motifs and conserved structural domains of PgWNK. Different color fonts represent different subfamilies, and different color boxes represent different conserved motifs and conserved structural domains

To elucidate the evolutionary and structural features of *WNK* genes in *P. ginseng*, we performed phylogenetic analysis of the encoded protein sequences and conducted motif/conserved domain prediction (Fig. 2). The phylogenetic clustering revealed six distinct subfamilies within the *PgWNK* family. Notably, subfamily VI exhibited the highest structural complexity, containing 12 conserved motifs compared to fewer motifs in other subfamilies. This divergence in motif composition suggests potential functional specialization among *WNK* subfamilies in *P. ginseng*. The results of the conserved structural domain analysis showed that *PgWNK09-13* and *PgWNK09-14* both belong to the PKc_like superfamily kinase protein, and subfamily VI has the PLN03150 superfamily conserved structural domain, whereas the other subfamilies all have the essential STKc_WNK superfamily structural domain.

### Chromosomal localization and gene duplication analysis of the *WNK* genes in *P. ginseng*

**Fig. 3:**
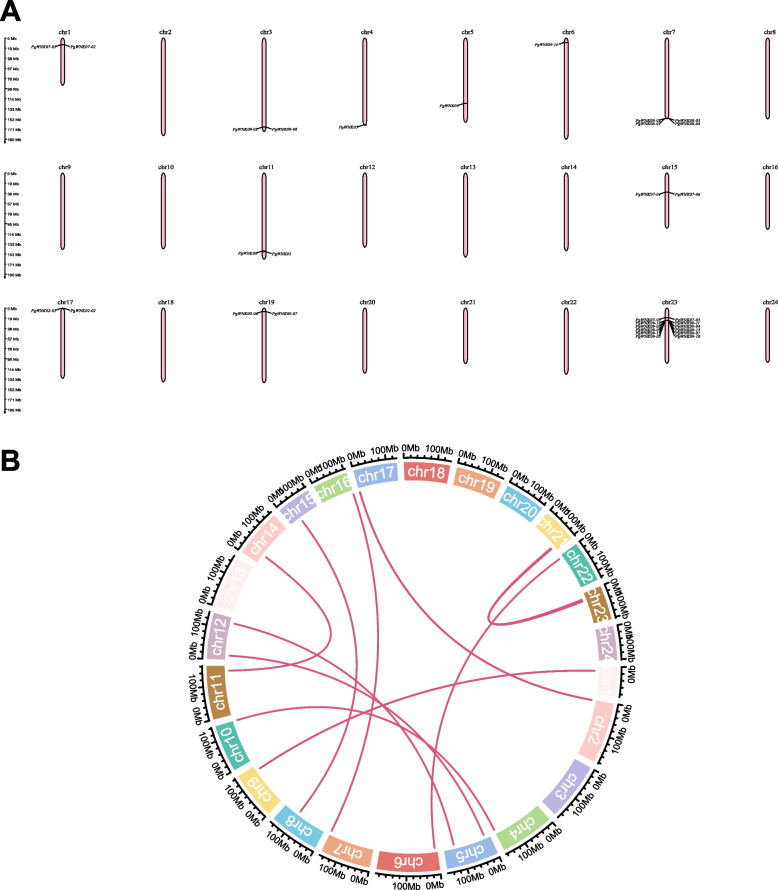
Chromosomal localization and collinearity analysis of the *WNK* gene family in *P. ginseng*. (A) Chromosome localization of the ginseng *WNK* gene family on 24 chromosomes. The chromosome number is represented at each bar top. (B) Co-linearity analysis of the *PgWNK* gene family members within the ginseng genome. The ends of the pink arcs point to parallel pairs generated by gene duplication, and the colored squares represent ginseng chromosomes, with the scale outside the chromosomes indicating the length of the chromosomes. Mb, megabase

Chromosomal localization analysis revealed that all 32 *WNK* genes were distributed across 24 chromosomes (chr2, chr8, chr9, chr10, chr12, chr13, chr14, chr16, chr18, chr20, chr21, chr22) of *P. ginseng* (Fig. 3 A). The distribution exhibited significant unevenness, with 13 chromosomes lacking detectable *PgWNK* family members, suggesting potential lineage-specific expansion or chromosomal loss events. But the 23rd chromosome contained the most *PgWNK* genes, with 12 of the 32 transcripts distributed on the 23rd chromosome (*PgWNK07-03*, *PgWNK09-12*, *PgWNK09-03*, *PgWNK09-09*, *PgWNK09-13*, *PgWNK09-05*, *PgWNK07-05*, *PgWNK09-11*, *PgWNK09-04*, *PgWNK09-15*, *PgWNK09-01*, and *PgWNK09-10*). According to the collinearity analysis diagram (Fig. 3B), there were 14 pairs of collinear *PgWNK* gene sequences connected, indicating gene duplication among members of the *WNK* gene family.

### GO annotation and functional differentiation of the *WNK* gene family

**Fig. 4:**
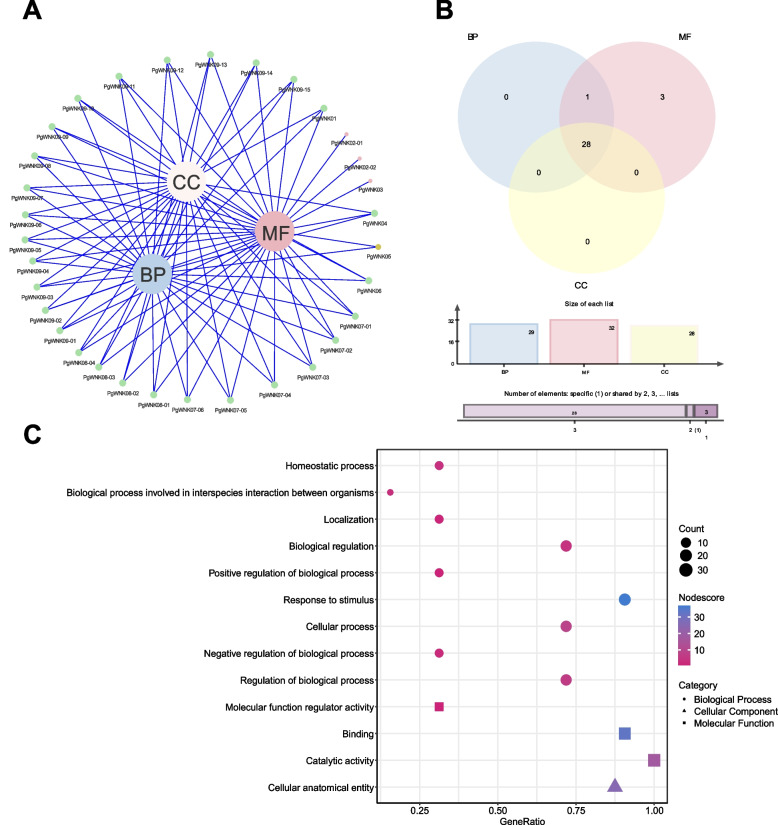
GO functional annotation and classification of *PgWNK* genes. (A) Network diagram of GO functional annotation of *PgWNK* genes. (B) Venn diagram of *PgWNK* genes in BP (biological process), MF (molecular function), and CC (cellular components) functional categories. (C) *PgWNK* genes were significantly enriched in GO function to 13 subcategories at GO level 2

We performed GO functional annotation of the 32 transcripts to understand the function of each *WNK* transcript. As shown in the Fig. 4 A, these 32 transcripts were annotated to three main GO functional categories, namely BP (biological process), MF (molecular function), and CC (cellular components). Among these 32 transcripts, 28 transcripts were annotated to all three functions of BP, MF, and CC; one transcript, *PgWNK05*, was annotated to both BP and MF functions, and the other three transcripts (*PgWNK02-01*, *PgWNK02-02*, and *PgWNK03*) were MF single-function annotated genes. This result indicates that there is a phenomenon of functional differentiation of the *PgWNK* gene family in ginseng. As shown in the Fig. 4B and Fig. 4 C, at level 2 of GO functional annotation, this gene family is annotated to different nodes under the three major categories: BP includes homeostatic processes (10), biological processes involved in interspecies interaction between organisms (5), localization (10), biological regulation (23), response to stimulus (29), cellular process (23), and regulation of biological process (23); MF includes molecular function regulator activity (10), binding (29), and catalytic activity (32); CC includes cellular anatomical entity (28), thus indicating that the *WNK* gene family has functional differentiation and plays a variety of functions in ginseng.

### Gene expression patterns analysis of the *PgWNK* genes

**Fig. 5:**
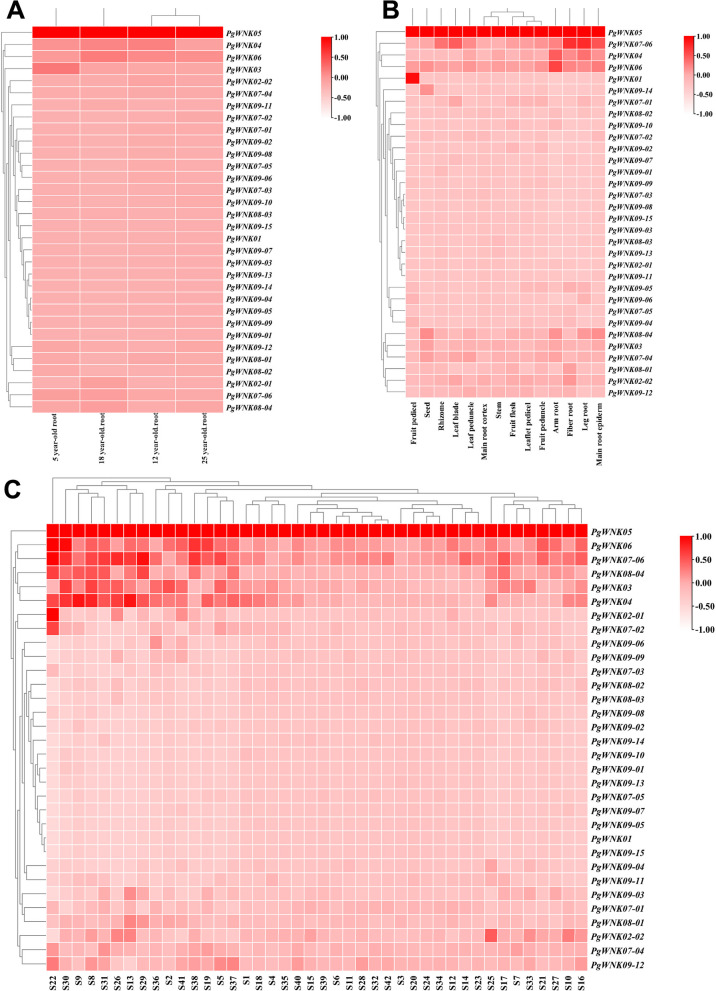
Heatmaps analysis spatiotemporal expression patterns of *PgWNK* genes in *P. ginseng*. (A) The *PgWNK* genes expressed in the 4 different ages (5, 12, 18, 25 years-old) of ginseng roots. (B) The *PgWNK* genes expressed in the 14 different tissues of 4-year-old ginseng. (C) The* PgWNK* genes expressed in the 42 farmer’s cultivars of 4-year-old ginseng roots

To investigate *WNK* genes expression patterns, we quantified *WNK *gene family abundance across four different ages, 14 tissues of four-year-old ginseng, and 42 local cultivars of ginseng roots in *P. ginseng*. The *PgWNK05* transcript showed the highest expression in the roots of ginseng at four different ages, in 14 tissue parts of four-year-old ginseng, and in 42 local cultivars. The *PgWNK01* transcript was highly expressed in the fruit pedicel of 14 tissue parts of four-year-old ginseng, and *PgWNK06*, *PgWNK07-06*, *PgWNK08-04*, *PgWNK03*, *and PgWNK04* these five transcripts showed relatively high expression in 42 local cultivars, indicating that the expression of *PgWNK* transcripts in ginseng varies with tissue, age, and local cultivar (Fig. 5).

### Co-expression network analysis of the *PgWNK* genes

**Fig. 6:**
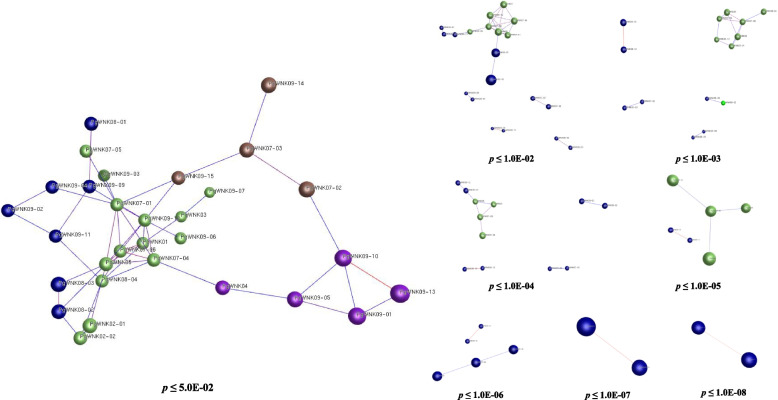
Co-expression network of *PgWNK* genes expressed in 42 local cultivars

Co-expression network analysis of 31 *PgWNK* transcripts (excluding *PgWNK01*, which showed no expression across 42 cultivars) revealed functional associations within *P. ginseng* (Fig. 6). Using an equivalent number of randomly selected non-WNK transcripts as reference controls. Co-expression network results showed *p* ≤ 0.05, 30 transcripts formed an interactive network (30 nodes, 50 edges), while under increasingly stringent thresholds (*p* ≤ 1.0E-08), the *PgWNK09-10* gene always maintained a strong correlation with the *PgWNK09-13* gene.

### Promoter cis-acting element analysis of the *PgWNK* gene family

**Fig. 7:**
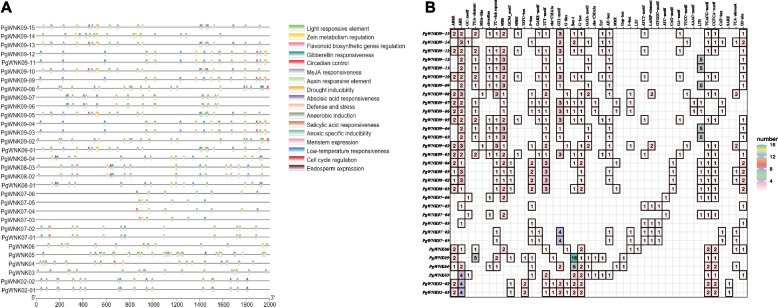
Promoter cis-acting element analysis of the *WNK* gene family in *P. ginseng*. (A) Cis-acting elements analysis of the *PgWNK* gene family. The different colors circles show cis-acting elements with different functions. (B) Heatmap analysis of cis-acting regulatory elements *PgWNK* genes

Cis-acting elements play a decisive role in the ginseng *WNK* gene family. In our study, 17 types of cis-acting elements were identified in the promoter region of the ginseng *WNK* gene family (Fig. 7 A). Among the identified elements, 32 *WNK* genes all contained light response and stress response cis-acting elements, and other identified elements in the promoter included endosperm expression elements, maize alcohol soluble protein metabolism regulatory elements, and meristem expression elements. The variation in the type and number of elements indicates that the functional variability of *WNK* genes in ginseng is large. As shown in the Fig. 7B, most *WNK* genes also contain various cis-acting elements related to hormone responses. Hormone response elements were divided into five categories: MeJA response, salicylic acid responsiveness, auxin-responsive elements, gibberellin responsiveness, and abscisic acid responsiveness. Twenty genes contained MeJA response elements, totaling 48 cis-acting elements; 11 genes contained salicylic acid responsive response elements, totaling 13 cis-acting elements; 14 genes contained auxin response elements, totaling 23 cis-acting elements; 23 genes contained gibberellin response elements, totaling 34 cis-acting elements, and 8 genes contained abscisic acid response elements, totaling 33 cis-acting elements. Cis-regulatory element analysis revealed a predominance of MeJA responsive motifs among hormone-related regulatory sequences, suggesting the *WNK* gene family in *P. ginseng* potentially mediates central regulatory functions in jasmonate-mediated signaling pathways.

### Screening of *WNK* candidate genes related to ginsenoside synthesis

To explore the function and role of *PgWNK* genes in the ginsenosides biosynthesis in ginseng under MeJA regulation, we established a co-expression network between *PgWNK* genes and known key enzyme genes involved in the biosynthesis of ginsenosides in ginseng, and analyzed the relationship between 20 *PgWNK* genes containing MeJA response elements and 20 known key enzyme genes involved in the biosynthesis of ginsenosides in ginseng. Five candidate *PgWNK* (*PgWNK05*, *PgWNK06*, *PgWNK07-04*, *PgWNK07-06*, and *PgWNK08-04*) genes were found to be closely related to five key enzyme genes involved in ginsenoside biosynthesis (*PgAS_6*, *PgSE2_1*, *PgSE2_4*, *PgSS_1*, and *PgUGT71 A27_2*), and as the *p* value decreased, these 10 genes still formed a cluster. We speculate that these 10 genes regulate the biosynthesis of ginsenosides in ginseng in a synergistic manner. Therefore, we selected these five candidates *PgWNK* genes for *WNK* gene expression and ginsenoside biosynthesis-related analyses under MeJA treatment.

### Expression analysis of *WNK* genes under MeJA treatment

**Fig. 8:**
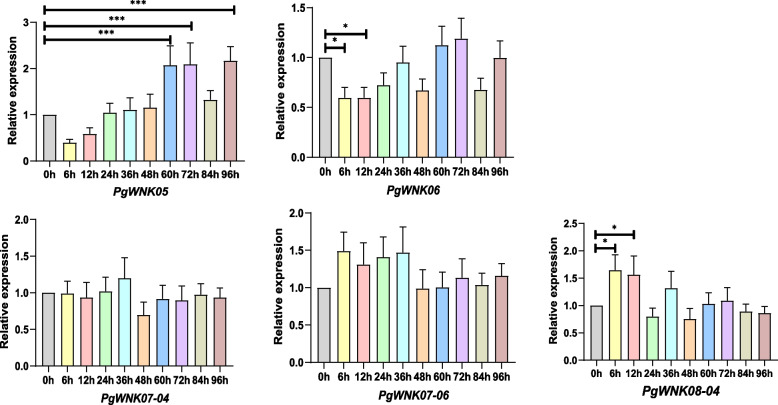
The expression of five *PgWNK* genes was detected under MeJA treatment of ginseng adventitious roots. The gene expressions of *PgWNK05*, *PgWNK06*, *PgWNK07-04*, *PgWNK07-06* and *PgWNK08-04* genes were analyzed by qRT-PCR at different treatment times. The gene expression at 0 h was set as"1"to calculate the relative expression of the five genes, values were the average of three replicates. “*” indicated significant difference at *p* ≤ 0.05, “**” indicated significant difference at *p* ≤ 0.01, “***” indicated significant difference at *p* ≤ 0.001, respectively

**Fig. 9:**
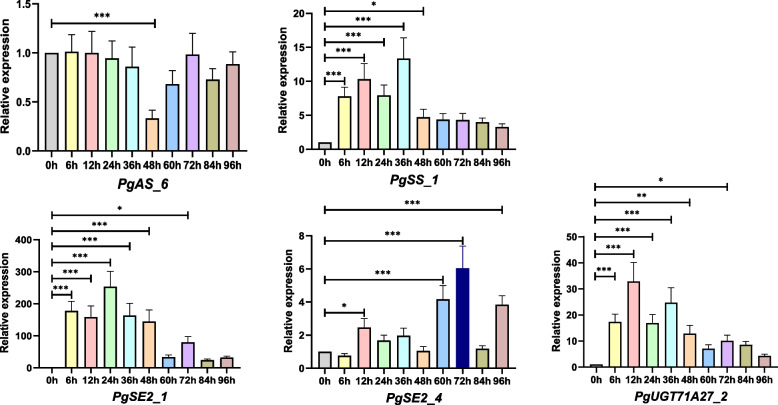
The expression of five key enzyme genes related to ginsenoside synthesis was detected under MeJA treatment of ginseng adventitious roots. The gene expressions of *PgAS_6*, *PgSE2_1*, *PgSE2_4*, *PgSS_1* and *PgUGT71 A27_2* genes were analyzed by qRT-PCR at different treatment times. The gene expression at 0 h was set as"1"to calculate the relative expression of the five genes, values were the average of three replicates. “*” indicated significant difference at *p* ≤ 0.05, “**” indicated significant difference at *p* ≤ 0.01, “***” indicated significant difference at *p* ≤ 0.001, respectively

After MeJA treatment, the expression of five *PgWNK* genes (*PgWNK05*, *PgWNK06*, *PgWNK07-04*, *PgWNK07-06*, and *PgWNK08-04*) and five key enzyme genes (*PgAS_6*, *PgSE2_1*, *PgSE2_4*, *PgSS_1*, and *PgUGT71 A27_2*) in ginseng adventitious root samples were detected at different time points. The results showed that these 10 genes could respond to MeJA induction to varying degrees (Fig. 8 and Fig. 9), three *PgWNK* genes (*PgWNK05*, *PgWNK06*, and *PgWNK08-04*), and five key enzyme genes could significantly respond to MeJA induction signals. The relative expression of *PgWNK05* (*p* ≤ 0.001) showed an upward trend within 72 h, but after a downward trend at 84 h, it quickly reached a peak at 96 h. The relative expression of the *PgWNK06* (*p* ≤ 0.05) was lower than the 0 h control group before 48 h, showing an upward trend at 60 h, but after 84 h, the relative expression was lower than that in the 0 h control group. The relative expression of *PgWNK08-04* (*p* ≤ 0.05) was significantly higher than that of the 0 h control group at 12 h but lower than that of the 0 h control group at 24 h (Fig. 8). The relative expression of the key enzyme genes *PgSS_1*, *PgUGT71 A27_2*, and *PgSE2_1* (*p* ≤ 0.05 and *p* ≤ 0.001) was higher than that in the 0 h control group within 96 h. The relative expression of *PgAS_6* (*p* ≤ 0.001) was almost the same as that in the control group within 36 h of MeJA induction, significantly decreased to the lowest level at 48 h, and then increased. The relative expression of the *PgSE2_4* (*p* ≤ 0.001) was higher than that in the 0 h control group after 6 h of induction and reached a peak at 72 h (Fig. 9). The results showed that the expression pattern of *PgWNK05* was the same as that of the four key enzyme-encoding genes.

### Correlation analysis between *PgWNK* gene expression changes and protopanaxatriol-type ginsenoside content under MeJA treatment

**Fig. 10:**
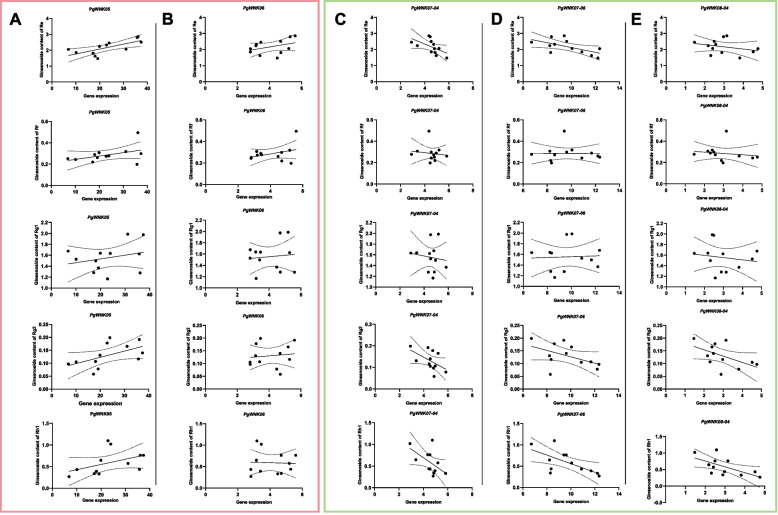
Correlation analysis between the relative expression levels of five *PgWNK* genes and five protopanaxatriol-types ginsenosides content (Re, Rf, Rg1, Rg2, and Rh1) induced under MeJA treatment. (A) Correlation between the *PgWNK05* expression and ginsenosides content. (B) Correlation between the *PgWNK06* expression and ginsenosides content. (C) Correlation between the *PgWNK07-04* expression and ginsenosides content. (D) Correlation between the *PgWNK07-06* expression and ginsenosides content. (E) Correlation between the *PgWNK08-04* expression and ginsenosides content

We performed a horizontal comparison and analysis of the expression of five *PgWNK* genes and the protopanaxatriol-type ginsenosides content under methyl jasmonate induction, and the correlation between the expression of the five *PgWNK* genes and the five protopanaxatriol-types ginsenosides content of Re, Rf, Rg1, Rg2, and Rh1 produced during induction was statistically analyzed (Fig. 10). As shown in Fig. 10 A, overall, as the relative expression of the *PgWNK05* gene increased, the content of the five saponins also showed an increasing trend, and the overall increase in the content of the five protopanaxatriol-type ginsenosides indicated a positive correlation between the two, that is, under the induction of methyl jasmonate, the increase of *PgWNK05* and the increase of saponin content; as shown in Fig. 10B, the content of four protopanaxatriol-type ginsenosides overall increased with the increase of the relative expression of the *PgWNK06* gene, only Rh1 decreased with the increase of the relative expression of the *PgWNK06* gene; as shown in Fig. 10 C and E, overall, as the relative expression of *PgWNK07-04* and *PgWNK08-04* genes increased, the content of the five saponins showed a downward trend, therefore, there was a negative correlation between the two, that is, as the *PgWNK07-04* and *PgWNK08-04* genes decreased, the saponin content increased; as shown in Fig. 10D, under MeJA induction, the relative expression of *PgWNK07-06* was negatively correlated with Re, Rg2, and Rh1, but the changes in the content of Rf and Rg1 saponins were not so significant. Overall, it was found that the expression of *PgWNK05* could affect the increase in the content of five monomer saponins with an increase in treatment time and was consistent with the expression pattern of four key enzyme genes, preliminarily inferring that the *PgWNK05* gene could synergistically interact with key enzyme genes to participate in the biosynthesis of protopanaxatriol-type ginsenosides. *PgWNK05* is also an important candidate gene for future studies on the function of *WNK* genes in ginseng.

## Discussion

*P. ginseng*, a plant of the *Araliaceae* family, is a medicinal and edible Chinese herbal medicine [33]. It has a history of thousands of years of use in our country and has broad application prospects and economic value [34]. In addition to responding to the action of plant hormones, the *WNK* gene family has been shown to regulate flowering time by regulating the photoperiod pathway [5], thereby participating in plant light signal transduction and responses to biotic and abiotic stresses [2].

We screened and obtained 32 *WNK* transcripts from the ginseng genome and transcriptome databases. Compared with other dicotyledonous plants sunflower (*H. annuus*), monocotyledonous plant rice (*O. sativa*) [7], and *A. calamus* [35], model plant *A. thaliana*, and spore plant *M. polymorpha* of the liverwort family [36]. The expanded *WNK* gene family members in *P. ginseng* likely reflects its tetraploid genome architecture and recurrent gene duplication events through evolutionary history, potentially attributable to whole-genome duplication mechanisms common in polyploid plants. Chromosome localization analysis showed that the distribution of the *WNK* gene family transcripts on ginseng chromosomes was uneven. Gene duplication plays a key role in the expansion of gene families [37, 38], and collinearity analysis results indicate that gene recombination events have occurred among *WNK* genes during the evolutionary derivation of ginseng. The results of GO functional annotation showed that among the 32 transcripts, 29 (90.6%) were annotated as metabolic processes in biological processes, indicating that *PgWNK* genes may play an effective role in the metabolic processes of ginseng. In terms of molecular functions, all 32 transcripts (100%) were annotated to binding functions, implying that the *PgWNK* family in ginseng mainly plays a role in DNA binding. These findings demonstrate functional diversification within the *PgWNK* gene family across its evolutionary trajectory.

The expression patterns of *PgWNK* genes results showed that the expression of *WNK* transcripts in the roots of ginseng at four different ages had time specificity; some transcripts were only expressed in the roots of ginseng at four different ages, and the expression of some transcripts showed an accumulation phenomenon with an increase in years. In 14 different ginseng tissues, the expression of *WNK* transcripts was the highest in the fruit peduncle of ginseng, but the gene expression was relatively high in the seeds, and the tissue-specific expression revealed that the *PgWNK* genes may play different functions in different tissue. Comparative analysis of three expression heatmaps (Fig. 5), we can be seen that the expression patterns of the same gene splicing-formed transcripts in the roots of ginseng in the same year, the same tissue, and in different local cultivars was different.

Promoter cis-regulatory elements are related to the potential biological functions of genes [39, 40], and through the analysis of cis-regulatory elements, it was found that members of the *WNK* gene family in ginseng have a wide range of biological functions. Cis-acting element analysis found that the *WNK* gene family potentially mediates multifaceted biological functions in *P. ginseng*, including defense mechanisms and systemic adaptation to abiotic stresses (drought, hypothermia, and so on) and circadian entrainment. The enrichment of hormone-responsive cis-elements in promoter regions strongly suggests its regulatory integration into phytohormone signaling cascades, particularly MeJA response. MeJA has been functionally validated as a potent biosynthetic elicitor, demonstrating significant capacity to upregulate ginsenoside production in vitro cell cultures and adventitious root systems [41]. Ginsenosides are the main active components extracted from ginseng, have a wide range of pharmacological activities, and have great economic value [42]. In our study, we used methyl jasmonate to treat the ginseng adventitious roots to investigate the *PgWNK* gene affects the ginsenosides biosynthesis in response to the jasmonate signaling pathway, we found the gene expression of *PgWNK* after MeJA treatment and found that the expression related to ginsenoside biosynthesis was significantly increased as the regulation time was prolonged. The expression of *PgWNK0*5 and *PgWNK06* genes increased over time, whereas that of *PgWNK08-04* gene decreased as the regulatory time increased. We also found that the expression of the *PgWNK05* gene increased with time after MeJA treatment, and the content of five protopanaxatriol-type ginsenosides Rb1, Re, Rf, Rg2, and Rh1 also showed an upward trend with the increase in gene expression, while the expression of *PgWNK08-04* decreased with time, and the content of five protopanaxatriol-type ginsenosides showed a downward trend. The expression pattern of *PgWNK05* was similar to those of the key enzyme genes *PgSE2_1*, *PgSE2_1*, *PgSS_1*, and *PgUGT71 A27_2*. Therefore, when studying the functions of *PgWNK05* and *PgWNK08-04* further, different genetic transformation strategies should be selected for in-depth research. While this study reveals the potential roles of the *WNK* gene family in ginsenoside biosynthesis, further studies are needed to elucidate the functions and molecular mechanisms of the *PgWNK05* candidate gene in ginsenoside biosynthesis.

## Conclusion

In this study, we systematically identified 32 *WNK* transcripts corresponding to nine *PgWNK* genes in *P. ginseng*. This study encompasses a comprehensive characterization analysis of this gene family, including phylogenetic tree, structural domain architecture, chromosomal distribution, collinearity relationships, GO functional annotation, expression pattern, co-expression networks, and cis-regulatory element analysis. Meanwhile, *PgWNK* candidate genes that significantly responded to MeJA were screened to regulate the increase in ginsenoside content under MeJA treatment, and the transcriptional dynamics of these candidate genes were time-dependent. Notably, *PgWNK05* was significantly upregulated and had significant regulatory effects on protopanaxatriol-type ginsenoside biosynthesis. These results establish the functional research for *WNK* genes in *P. ginseng*, providing critical insights into their roles in secondary metabolism and jasmonate-mediated regulation.

## Data Availability

The datasets generated and analysed during the current study are available in the National Center for Biotechnology Information (NCBI), repository, BioProject PRJNA302556; and at Gene Expression Omnibus (GEO) of NCBI, SRP066368 and SRR13131364—SRR13131405. The plant materials are available through corresponding authors, upon request.

## Declarations

### Supplementary Information

Supplementary material 1: Table S1. Basic information of the *WNK* gene family in *Panax ginseng*.
